# Autonomic responses to emotional linguistic stimuli and amplitude of low-frequency fluctuations predict outcome after severe brain injury

**DOI:** 10.1016/j.nicl.2020.102356

**Published:** 2020-07-21

**Authors:** Gerardo Salvato, Manuela Berlingeri, Gabriele De Maio, Francesco Curto, Arturo Chieregato, Francesca Giulia Magnani, Maurizio Sberna, Mario Rosanova, Eraldo Paulesu, Gabriella Bottini

**Affiliations:** a Cognitive Neuropsychology Centre, ASST “Grande Ospedale Metropolitano” Niguarda, Milano, Italy; b Department of Brain and Behavioral Sciences, University of Pavia, Pavia, Italy; c NeuroMi, Milan Center for Neuroscience, Milano, Italy; d Department of Humanistic Studies, University of Urbino Carlo Bo, Urbino, Italy; e Center of Developmental Neuropsychology, Area Vasta 1, ASUR Marche, Pesaro, Italy; f Department of Neuroresuscitation and Intensive Care, ASST “Grande Ospedale Metropolitano” Niguarda, Milano, Italy; g Department of Neuroradiology, ASST “Grande Ospedale Metropolitano” Niguarda, Milano, Italy; h Department of Biomedical and Clinical Sciences “Luigi Sacco”, University of Milano, Italy; i Fondazione Europea di Ricerca Biomedica, FERB Onlus, Milano, Italy; j Psychology Department and NeuroMI-Milan Center for Neuroscience, University of Milano-Bicocca, Milan, Italy; k fMRI Unit, I.R.C.C.S. Galeazzi, Milano, Italy

**Keywords:** Consciousness, MRI, Skin conductance, Language, fALFF.

## Abstract

An accurate prognosis on the outcome of brain-injured patients with disorders of consciousness (DOC) remains a significant challenge, especially in the acute stage. In this study, we applied a multiple-technique approach to provide accurate predictions on functional outcome after 6 months in 15 acute DOC patients. Electrophysiological correlates of implicit cognitive processing of verbal stimuli and data-driven voxel-wise resting-state fMRI signals, such as the fractional amplitude of low-frequency fluctuations (fALFF), were employed. Event-related electrodermal activity, an index of autonomic activation, was recorded in response to emotional words and pseudo-words at baseline (T0). On the same day, patients also underwent a resting-state fMRI scan. Six months later (T1), patients were classified as outcome-negative and outcome-positive using a standard functional outcome scale. We then revisited the baseline measures to test their predictive power for the functional outcome measured at T1. We found that only outcome-positive patients had an earlier, higher autonomic response for words compared to pseudo-words, a pattern similar to that of healthy awake controls. Furthermore, DOC patients showed reduced fALFF in the posterior cingulate cortex (PCC), a brain region that contributes to autonomic regulation and awareness. The event-related electrodermal marker of residual cognitive functioning was found to have a significant correlation with residual local neuronal activity in the PCC. We propose that a residual autonomic response to cognitively salient stimuli, together with a preserved resting-state activity in the PCC, can provide a useful prognostic index in acute DOC.

## Highlights

• Acute DOC patients with favourable outcome show preserved event-related electrodermal response.
• Acute DOC patients showed reduced fALFF in the posterior cingulate cortex.
• Event-related electrodermal activity correlated with the fALFFs in the PCC in the acute phase.

## 1 Introduction

Anoxic, haemorrhagic, or traumatic brain injuries may result in disorders of consciousness (DOC). At the acute stage, these disorders are characterised by the impairment of the two main aspects of consciousness, i.e., wakefulness and awareness (Laureys, 2005). DOC are commonly assessed in the clinical practice using such tools as the Coma Recovery Scale–Revised (CRS-R; Giacino et al., 2004), which is primarily based on the patients’ behavioural responsiveness to stimuli. However, several aspects of awareness could be preserved in chronic DOC patients in the absence of an explicit behavioural interaction with the environment (Andrews et al., 1996, Childs et al., 1993, Demertzi et al., 2008, Eickhoff et al., 2008, Fernandez-Espejo et al., 2008, Giacino et al., 2009, Majerus et al., 2005, Monti et al., 2009, Owen et al., 2002, Owen et al., 2006, Owen et al., 2005, Rosanova et al., 2012, Schnakers et al., 2009). For instance, in a seminal study, Owen and colleagues (2006) used a motor imagery paradigm during fMRI to detect the neural correlates of command-following in one patient diagnosed as behaviourally vegetative. Since then, several studies have replicated this finding in similar chronic patients (see also the recent *meta*-analysis by Berlingeri et al., 2019 for further details). For example, Monti and colleagues (2010) studied a group of 54 patients using a similar fMRI paradigm. They found that only 5 patients were able to modulate their brain activity wilfully. In three of these patients, additional bedside clinical assessment revealed some signs of awareness. One patient was able to use their technique to answer yes or no to questions during the fMRI experiment. Moreover, Cruse et al., 2011, Cruse et al., 2012 expanded these results further using an EEG task involving motor imagery to detect command following responses at patients’ bedsides. However, the adoption of unimodal neuroimaging markers for both diagnosis and prognosis of DOC in clinical routine is still debated. According to the most recent guidelines of the American Academy of Neurology (Giacino et al., 2018)*,* there is currently insufficient evidence to support or refuse the routine clinical use of functional neuroimaging (fMRI, EEG, PET) in DOC, and available results fail to outline a clear-cut picture (Berlingeri et al., 2019). Nonetheless, multimodal evaluations incorporating specialised functional imaging or electrophysiological indices are considered promising as they might reduce the risk of misdiagnosis associated with neurobehavioural assessments (Giacino et al., 2018). Accordingly, one of the primary aims of the research agenda is to develop prognostic approaches based on multiple and complementary techniques (Cavaliere et al., 2018, Coleman et al., 2009, Giacino et al., 2014, Gibson et al., 2014, Gosseries et al., 2014, Owen et al., 2009).

One of the main challenges facing multimodal brain-based assessments in acute DOC patients is the routine use of sedation in this population. An example of this is provided by a recent study by Edlow et al. (2017). The authors studied 16 acute DOC patients using multiple task-based fMRI and EEG protocols. They found a cortical response for bottom-up language, music stimulation, and motor imagery in four out of 16 patients, although these patients did not present a significant association between the functional MRI data and the results of the Glasgow Outcome Scale-Extended (GOS-E; Wilson et al., 1998). Unfortunately, most of the patients included in their study (8/16) were under propofol sedation during the EEG or fMRI tasks. Only one out of these eight patients showed cognitive motor dissociation, although the authors reported that the type of sedation was not associated with functional MRI/EEG responses or level of consciousness at the time of assessment. A more extreme case was presented by Huang et al. (2018), where one out of five participants exhibited specific fMRI signatures of volitional mental imagery while behaviourally unresponsive due to propofol sedation, making the interpretation of findings in DOC patients under sedation, if anything, more complicated.

The need for approaches based on multiple and complementary techniques to develop reliable prognostic markers received further support from a recent multi-dimensional prognostic model capable of predicting outcome after one year (Song et al., 2018). The model was based on a combination of resting-state fMRI measures, aetiology, onset age, and DOC duration. However, the study of Song et al. (2018) was based on patients tested at varying time intervals from the onset, mostly far later than the acute stage (i.e., when a prognostic evaluation is crucial). Furthermore, the proposed model lacks indicators of residual cognitive activity in the patient population.

To summarise, there is encouraging evidence that a combination of imaging techniques using a variety of tasks could support the evaluation of DOC. However, suitable protocols that may help to formulate a reliable prognosis that can be adopted easily in the clinical setting (e.g., bedside evaluation tools or basic imaging techniques) are still to be identified. Here, we report our efforts to improve the outcome predictions in acute DOC patients. A bedside event-related Skin Conductance Response (SCR) paradigm that addresses the differential autonomic responses to emotionally salient words as compared to pseudo-words was employed in the early-stage DOC patients (16.6 ± 9.5 days from the onset). We combined event-related SCR with structural MRI and resting-state fMRI markers with the following aims: i) to test the possibility of detecting early signs of residual cognitive functioning in response to linguistic stimuli, ii) to investigate the prognostic value of such responses, and iii) to correlate the electrodermal prognostic marker with entirely data-driven voxel-wise measures of resting-state fMRI (i.e., the fractional amplitude of low-frequency fluctuations (fALFF) a known marker of preserved neurofunctional activity that can be used to detect cerebral regions with abnormal local functioning (Chen et al., 2015, Zou et al., 2008). Patients were followed up 6 months later (T1) with a standardised functional outcome measure (GOS-E) that allowed us to classify them in terms of a positive or negative outcome. According to our hypotheses, we expected an earlier differential physiological response to emotionally salient words compared to pseudo-words in outcome-positive patients. The physiological response should also correlate with the residual power of local neuronal activity in regions involved in the regulation of autonomic responses to external stimuli and, arguably, in critical nodes of the so-called “Consciousness Network” (Vogt and Laureys, 2005).

## 2 Materials and methods

### 2.1 Event-related physiological study and patients classification

#### 2.1.1 Participants

Patients were recruited at the Department of Neuroresuscitation and Intensive Care of the “ASST Grande Ospedale Metropolitano" Niguarda in Milan from 2016 to 2018. Only patients who met the following inclusion criteria were enrolled in the study: i) age range between 18 and 80 years old; ii) native Italian speakers (or highly-proficient bilingual in one case); iii) absence of previous psychiatric, neurological, or drug abuse history; iv) free from sedatives and anxiolytic and analgesic medication for at least 5 days (according to the patient’s pharmacological therapy and physical biometric data); v) right-handedness; vi) no hearing deficits documented by the evoked potentials; and vii) no contraindications for the MRI exam. From a cohort of 1535 patients’ admissions, 31 subjects were tested. Fifteen right-handed DOC patients (6 males) between 43 and 77 years of age (*M* = 63.9, *SD* = 8.3) met the inclusion criteria and were enrolled (clinical details in Supplementary Table 1). Thirty-five healthy, right-handed adults (16 males) between 21 and 74 years of age (*M* = 33.2, *SD* = 14.9) were also enrolled. All participants were native Italian speakers, had normal hearing, and had no previous history of mental or neurological illness. The Ethical Committee Milano Area C approved the study (study protocol ID667). Healthy individuals and patients’ relatives gave written informed consent in accordance with the Declaration of Helsinki.

#### 2.1.2 Behavioural examination and outcome assessment

Immediately before administering the experimental paradigms (at T0), the patients’ level of consciousness was assessed using the Coma Recovery Scale-Revised (CRS-R). Functional outcome at 6 months (T1) was assessed using the GOS-E. Alternatively, patients or their surrogates were assessed through a validated GOS-E phone questionnaire (Pettigrew et al., 2003). In line with previous studies (Chennu et al., 2017, Stender et al., 2014), a GOS-E score threshold of two was used to classify patients as either “outcome-negative” (GOS-E score ≤ 2), or “outcome-positive” (GOS-E score > 2).

#### 2.1.3 Event-related skin conductance response

Participants received instructions as if they could fully understand and follow what the examiner said. Subjects were required to pay attention to 30 emotionally salient words and 30 pseudo-words presented through headphones in random order, with an onset-to-onset time interval of 12 s, while the skin conductance response (SCR) is recorded. Words were retrieved from a pool of stimuli classified according to their level of arousal and significance (Dindo and Fowles, 2008). The 30 words with the highest ranking of arousal and significance were selected from the list and were used to create 30 pseudo-words by changing the letter order of each word, thus maintaining word length and the number of syllables (see the list of stimuli in the Supplementary Table 3). As a final step, we checked that the obtained pseudo-word stimuli were not meaningful. Stimuli were recorded through a vocal synthesiser to avoid any familiarity effect due to the human voice. This ad hoc paradigm aimed to maximize the chance of detecting even the smaller electrodermal signs of lexical-semantic processing by contrasting the event-related SCRs elicited by emotional words (i.e., lexical entries with both a meaning and an emotional value), with event-related SCRs elicited by pseudo-words (i.e., verbal stimuli that do not have meaning or emotional value). Because the two categories of stimuli can be considered physically identical, we hypothesised that SCR differences during the processing of words and pseudo-words could be ascribed to lexical-semantic processing.

During the experiment, SCR was measured using the BIOPAC MP-150 Data Acquisition System (BIOPAC Systems, Inc., Goleta, CA) and the EDA-100C module. The electrodermal signal was recorded from two electrodes attached to the index and middle fingers of the participants’ non-dominant hand. SCR was measured with a sensitivity of 0–100 lS, the signal sampled at 500 Sa/s 16-bit, low-pass filtered at 5 Hz (Scott et al., 2011).

#### 2.1.4 Data pre-processing and analyses

For each trial, SCR was determined as the difference between the maximum value detected in 8-s post-stimulus and the baseline calculated as the average value of a 3-s pre-stimulus (peak-to-base measure*;* Rhudy et al., 2007, Romano et al., 2014). The peak-to-base measures were then normalised within-subjects and converted to z-scores to reduce the effect of the inter-subject variability of SCR, which is commonly large. The SCR signals extracted from the group of healthy controls and the group of patients were analysed separately. In the case of healthy controls, after ensuring that the data followed a normal distribution, we applied a paired-sample *t*-test to assess the physiological correlates of the implicit discrimination between words and pseudo-words. In a second step, we computed an SCR delta index for each participant, namely the SCR mean difference between the two classes of stimuli. This measure was used to investigate the predictive power of our task over the functional outcome. In particular, we first computed the median value of the SCR delta index in healthy controls, hypothesising that patients with residual lexical-semantic skills may present with a comparable physiological response. Due to the relatively small sample size of the outcome-negative patients’ group, we adopted a robust statistical approach based on non-parametric tests. The median value of the SCR delta index in healthy controls was taken as the reference value under the null hypothesis in a one-sample Wilcoxon Signed Ranked test, using SPSS 20 (Statistical Package for the Social Sciences, IBM Corp. Released 2011. IBM SPSS Statistics for Macintosh, Version 20.0. Armonk, NY: IBM Corp). We also compared outcome-positive and outcome-negative patients’ SCR delta indices. The frequentist analysis was supplemented with a Bayesian approach (JASP Team, 2018).

### 2.2 Neuroimaging study

#### 2.2.1 Participants

DOC patients also underwent an rs-fMRI scan. One patient did not participate due to technical problems, and the final sample included 14 participants (5 males, age range 43–77 years-old, *M* = 63.6, *SD* = 8.5). Fourteen healthy, age-matched controls (5 males, age range 43–72 years-old, *M* = 64.1, *SD* = 6.87) with no previous history of mental or neurological illness were also recruited.

This study was carried out in accordance with the recommendations of the Ethical Committee Milano Area C (study protocol ID667) with written informed consent from the participants. Healthy individuals and patients’ relatives gave written informed consent in accordance with the Declaration of Helsinki.

#### 2.2.2 Resting state imaging

MRI scans were performed by using a General Electrics 1.5 T *Signa* scanner equipped with gradient-echo echo-planar (Flip angle 90°, TE = 60 msec, TR = 3000 msec, FOV = 280 × 210 mm; matrix = 96 × 64). Each volume consisted of 35 contiguous oblique images (thickness = 4 mm, gap = 0 mm) acquired along the AC-PC plane. A total of 200 volumes were acquired (acquisition time = 10 min). During the fMRI scan, healthy participants and DOC patients were instructed to keep their eyes closed, not to move, and to avoid thinking about anything in particular. Moreover, for each participant, a T1-weighted anatomical scan was acquired using a 3D-SPGR sequence (Flip angle = 20°, TE = 2.92 ms, TR = 9.16 ms, acquisition matrix = 256 × 256, slice thickness = 1 mm, interslice gap = 0 mm, and voxel size = 1 × 1 × 1 mm). The volumetric MRI scan included 150 slices acquired on oblique sections parallel to the AC-PC line to cover the entire brain volume.

Although the eyes-closed resting condition can lead to participants falling asleep during scanning and to more variability among responses (Agcaoglu et al., 2019, Patriat et al., 2013, Tagliazucchi and Laufs, 2014), this choice was driven by methodological considerations. Firstly, DOC patients at the acute stage very rarely present with spontaneous eye-opening. Thus, we decided to apply this procedure to both healthy controls and patients to keep the experimental condition as homogeneous as possible. Secondly, in an eyes-open condition, brain-damaged patients, whose lesions could involve the attentional system, might explore space around them differently from healthy individuals, which could lead to confounding between-groups differences. Lastly, our approach is in line with the methodology of previous studies that have investigated fALFFs in DOC patients (He et al., 2014, Huang et al., 2014).

#### 2.2.3 Data pre-processing and analyses

The resting-state analyses were conducted using the DPARSF − Advanced Edition toolbox (DPARSF-A; http://rfmri.org/DPARSF). The fMRI scans were realigned, and the volumetric T1 image was coregistered to the functional ones. Once coregistered, the T1 image was passed to the unified segmentation algorithm (Ashburner and Friston, 2005) to obtain the grey, white, and cerebrospinal fluid maps. At this stage, signal decomposition was applied to partial out the effects of global signal, of the rigid body transformation (six parameters), and of white matter and cerebrospinal fluid signals. The fMRI images were then normalised to the MNI EPI template and rescaled to obtain isotropic voxels of 3 × 3 × 3 mm. After normalisation, we checked for the quality of warped images by using the “normalisation check” option in DPARSF-A. None of the patients showed significant deformations of the brain caused by potential problems during the preprocessing of the data when lesions are present. The normalised images were finally smoothed with a Gaussian FWHM filter of 4 × 4 × 4 mm.

After pre-processing, the signal was filtered to isolate low-frequency fluctuations within the 0.01–0.08 Hz frequency ranges that are thought to reflect the spontaneous neuronal activity. For each participant, a voxel-wise approach was employed to extract the mean time course for fluctuations in the selected frequency range from the entire brain volume. This allowed us to create subject-specific fractional Amplitude of Low-Frequency Fluctuations (fALFF) maps (Zou et al., 2008). fALFFs have been defined as the power within the low frequency range (0.01–0.1 Hz) divided by the total power in the entire detectable frequency range and, as a consequence, it represents the relative contribution of specific low-frequency oscillations to the whole frequency range. In particular, here we focused on the voxel-wise distribution of fALFF for three main reasons: i) the detection of regional neurofunctional abnormalities is crucial to clinical studies as it has been revealed as a powerful instrument to detect neurological (Egorova et al., 2017), psychiatric (Hoptman et al., 2010) and developmental pathologies (Zang et al., 2007); ii) the adoption of whole-brain voxel-wise analysis allowed us to explore between-groups differences without relying on any *a priori* hypothesis, due to the relative lack of evidence in the literature; iii) the adoption of the fractional measure allowed us to control for the physiological noise induced by cardiac and respiratory pulsations (Birn et al., 2006, Hu et al., 1995, Lund et al., 2006, Shmueli et al., 2007, Wise et al., 2004) on the one hand, and to avoid the confounding effect that is typically observed in the CSF with raw ALFF (Zou et al., 2008) on the other. The fALFF maps were z-transformed before the statistical analyses (zfALFF). Voxel-wise analyses were performed at the whole-brain level using the Statistical Parametric Mapping software (SPM12, Wellcome Department of Imaging Neuroscience, London, UK; http://www.fil.ion.ucl.ac.uk/spm/software/spm12/). In particular, we compared the voxel-wise amplitude of the zfALFF between the healthy controls and the patients (two-sample *t*-test); the contrasts were thresholded at *p* < 0.05 cluster-level FWE correction; this was followed by a *p* < 0.001 uncorrected at the voxel level (Worsley et al., 1996). The local maxima within the cluster were further explored by adopting a nested-taxonomy (Friston et al., 1996). From the neurofunctional point of view, this allowed us to depict the cluster extension of between-group differences better.

We used the thresholded map “Controls > Patients” obtained at *p < 0.001* (uncorrected) as a region of interest to explore the relationship between the SCR delta index and the amplitude of the low-frequency fluctuations in the patients’ group. The zfALFF maps were treated as a dependent variable and the skin-conductance SCR delta index as a predictor in a simple non-parametric regression using the SnPM toolbox (http://warwick.ac.uk/snpm). Results were displayed at *p < 0.05* FWE-corrected at the voxel level. Finally, the volumetric images (T1-weighted) were processed with the toolbox ALI (Automatic Lesion Identification; Seghier et al., 2008) to detect subject-specific brain lesions automatically. The resulting lesion maps were then passed to the “lesion overlap mapping” step available in ALI to obtain a comprehensive and quantitative map of the lesions detected in the entire sample of DOC patients (the results are reported in Fig. 3). The resulting lesion maps were visually inspected to confirm the validity of the automated process. This last step was performed to check whether the voxel-wise results of the between-groups comparison ran over the zfALFF maps that fell within damaged areas.

## 3 Results

### 3.1 Event-related physiological study

As a first step, we evaluated whether our experimental paradigm could be adopted to detect significant electrodermal differences between words and pseudo-words in healthy controls. The data passed the Kolmogorov–Smirnov test for normality (SCR words: *D*_(35)_ = 0.089; *p* = 0.200; SCR pseudo-words: *D*_(35)_ = 0.114; *p* = 0.200). Results showed a significant difference between the mean physiological response associated with words (SCR *Mean* = +0.15 *SD* = 0.11) and pseudo-words (SCR *Mean* =  − 0.16 *SD* = 0.13; *t*_(34)_ = 7.52; *p* < 0.001; Bayesian Factor *BF_10_* = 1.314e + 6, i.e., a very strong evidence for the alternative hypothesis). Moreover, it is worth noting that the delta value “words > pseudo-words” did not correlate with age (r_s(34)_ =  − 0.07, p = 0.698), gender (r_s(34)_ = 0.07, p = 0.671), and education (r_s(34)_ = 0.12, p = 0.501), providing further support for the generalisability of our approach. In the healthy group, the median value of the difference between the electrodermal activity generated from words and pseudo-words was 0.28. This value was taken as a reference point to evaluate the physiological responsiveness in the two samples of patients.

**Fig. 1:**
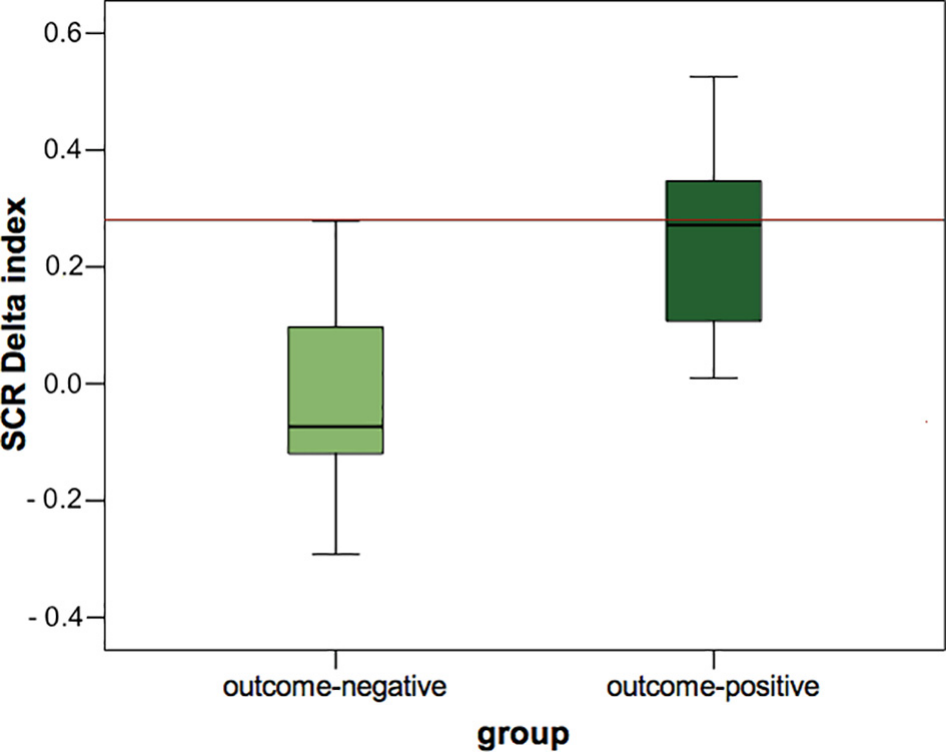
The figure shows the SCR delta index value (words > pseudo-words) of the outcome-negative and outcome-positive patients groups against the median value of the healthy control group that is represented by the red line (median = 0.28). (For interpretation of the references to colour in this figure legend, the reader is referred to the web version of this article.)

The prognostic value of the delta index extracted from our experimental paradigm was tested by classifying the patients in outcome-positive (N = 10) and outcome-negative (N = 5) according to the criteria reported in the previous section. Interestingly, the SCR results collected at T0 (i.e., in the acute phase) showed that the outcome-positive patients’ median value (median = 0.27) did not differ from the healthy controls’ one (Wilcoxon *Z* = -0.459; *p* = 0.646; *BF_10_* = 0.37, i.e., moderate evidence for the null hypothesis). Conversely, the Wilcoxon signed-rank test indicated that the outcome-negative patient’s median value (median = -0.07) was significantly lower than the healthy group’s median value (Wilcoxon *Z* = -2.023; *p* = 0.043; *BF_10_* = 2.80, i.e., moderate evidence for the alternative hypothesis; see Fig. 1). Furthermore, the Mann-Whitney *U* test computed to compare the delta values between outcome-positive and outcome-negative patient groups was significant (*Z* = -2.205; *p* = 0.028; *BF_10_* = 3.69, i.e., moderate evidence for the alternative hypothesis). When taken together, the results of the event-related physiological study suggest that it is possible to detect early signs of residual cognitive functioning in response to linguistic stimuli in acute DOC patients and that these signs may have a prognostic value.

### 3.2 Neuroimaging study

**Fig. 2:**
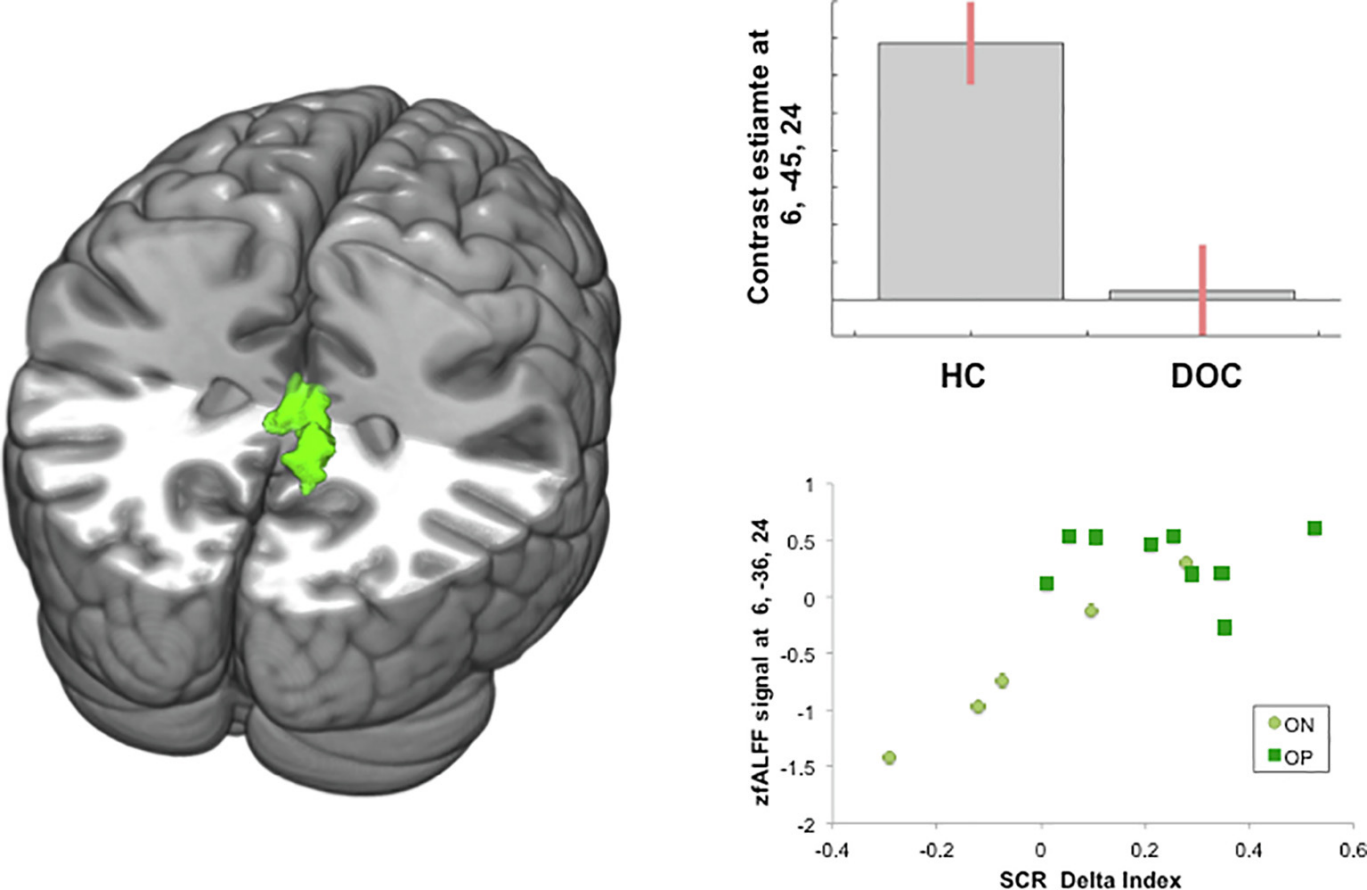
Left panel: The figure shows the results of the linear t-contrast “Healthy Controls > DOC Patients” (at *p* < 0.001 uncorrected voxel-wise; p < 0.05 FWE-corrected cluster level). Right panel: The upper graph shows the effect-size of the linear t-contrast “HC > DOC” in the PCC; the lower panel shows the relationship between the patients’ electrodermal responses (on the x-axis) and the level of zfALFF (on the y-axis) in the PCC: the higher the SCR Delta Index, the higher the zfALFF in the PCC (Spearman Rho = 0.54, p_one-tailed_ = 0.02). Here it is worth noting that the circles in light green represent outcome-negative (ON) patients, and the squares in dark green depict outcome-positive (OP) patients. (For interpretation of the references to colour in this figure legend, the reader is referred to the web version of this article.)

As a first step, we isolated the voxel-wise between-groups differences in the level of zfALFF. The direct comparison between healthy controls and DOC patients showed a significant between-group difference in a cluster centred in the right posterior cingulate cortex (PCC: *x* = 6, *y* = −45, *z* = 24, *Z-score* = 5.0, *p* < 0.05 *FWE*-corrected cluster level). In the same cluster, we also found a reduction of the amplitude of low-frequency fluctuations in the homologue area of the left hemisphere (extending from the midline *x* = 0, *y* = –33, *z* = 24 to *x* = −6, *y* = −40, *z* = 22 and *x* = −3, *y* = −38, *z* = 24), and in the right precuneus (see Table 1 and Fig. 2). The reverse contrast, namely “DOC patients > Controls,” did not yield any significant result, even at a less conservative threshold.

**Table 1:** Brain regions (MNI coordinates) that showed a significant reduction of the zfALFFs in DOC patients (i.e., linear t-contrast “healthy controls > DOC patients” thresholded at p < 0.001 uncorrected voxel-level, p < 0.05 FWE-corrected cluster level). The asterisk indicated that the result also survived the most conservative thresholding method available in SPM (i.e., at the p < 0.05 FWE voxel-level corrected).

	*MNI Coordinates*							
	*x*	*y*	*z*	*Z score*	*x*	*y*	*z*	*Z score*
Brain regions	Left hemisphere				Right hemisphere			
Posterior Cingulate Gyrus	0	–33	24	4.51	6	−45	24	5.02*
Precuneus					9	−54	24	3.95

**Fig. 3:**
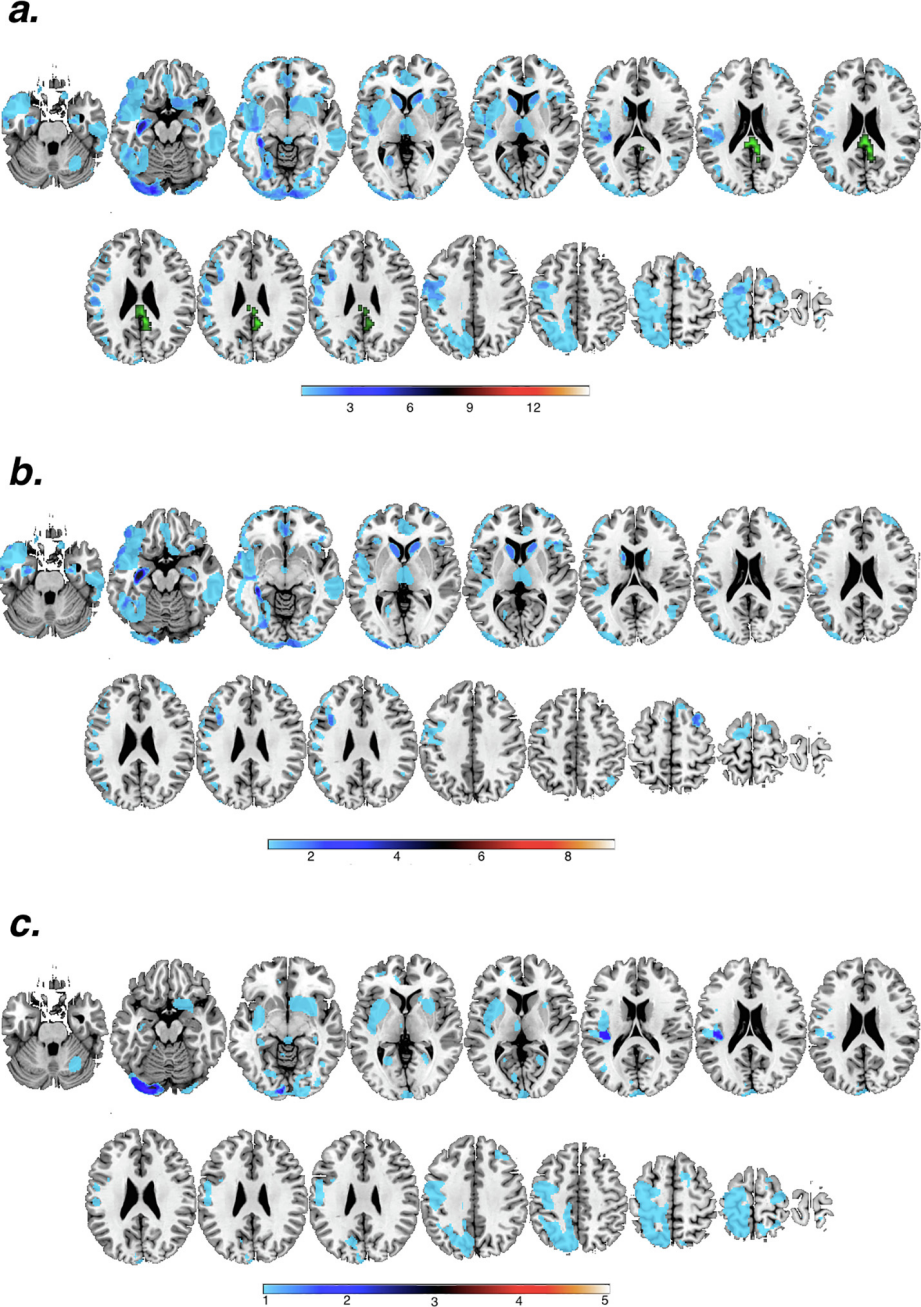
Panel “a” shows the neuroanatomical distribution of the lesions in the entire sample of patients (n = 14; results extracted from the analysis performed with the ALI toolbox). The region in green corresponds to the cluster centred in the posterior cingulate cortex (*x* = 6; *y* = -45; *z* = 24) to show that this region is anatomically preserved in the entire sample of DOC patients. Panel “b” and “c” show the neuroanatomical distribution of the lesion in the outcome-positive and outcome-negative patient groups, respectively. (For interpretation of the references to colour in this figure legend, the reader is referred to the web version of this article.)

The result of the between-group comparison was further explored by adopting a non-parametric approach to test whether the electrodermal prognostic marker (i.e., the delta index that was extracted from the event-related physiological study) correlated with spontaneous neural activity. To this end, the SCR delta index was entered as a predictor in a non-parametric regression analysis. The results showed a positive relationship between the SCR delta index and the zfALFF signal of the PCC (*x* = 6, *y* = −36, *z* = 24, *p* < 0.05 FWE-corrected voxel-level; Spearman Rho = 0.55, p_one-tailed_ = 0.02; Fig. 2). Furthermore, a positive relationship was observed between the SCR delta index and GOS-E scores (Spearman Rho = 0.56, p_two-tailed_ = 0.031).

Interestingly, none of these effects fell within the lesion overlap map (see Fig. 3), to further suggest that a spurious effect of brain lesions did not bias our findings (see also supplementary materials for additional analyses).

## 4 Discussion

Severely brain-injured patients with DOC represent a major challenge concerning prognosis and consequent daily care. Innovative multiple-technique approaches that are independent of patients’ overt behavioural responses may provide useful information regarding the outcome. To better explore residual lexical-semantic processing of emotionally salient linguistic stimuli in DOC patients, we implemented a simple event-related SCR paradigm based on implicit discrimination of auditorily presented words and pseudo-words. Only the group of patients with a positive functional outcome after six months presented an electrodermal-evoked response for the emotionally salient words comparable to those of healthy controls at baseline.

Few studies combined electrodermal response recording with the presentation of auditory stimuli in DOC patients, and most of them included chronic DOC patients (see Supplementary Table 2 for further details). In five out of the seven available studies, low-level stimuli were employed (e.g., electrical stimuli). These simple stimuli might have elicited automatic responses unrelated to higher-level cognitive processing. Furthermore, no available studies reported a follow-up of the patients’ functional outcomes after the initial assessment. For example, in the study by Daltrozzo and colleagues (2010), event-related SCR associated with the presentation of emotional and non-emotional sounds were collected in a sample of 13 low-responsive (min_GCS_ = 4, max_GCS_ = 6) acute (mean days from brain damage = 3.9) patients. In this sample, as in the healthy control group, a significant SCR effect was detected in the emotional vs. neutral sound condition. However, the relative prognostic value of their findings was not reported with follow-up data.

Of note, meaningful fMRI results from an entirely data-driven index mirrored the electrodermal pattern in our study. In particular, we found that DOC patients had a significant zfALFF reduction clustered in the retrosplenial cortices, a pivotal region for conscious information processing (“Consciousness Network”; Vogt and Laureys, 2005), and a well-known hub of the Default Mode Network (Fransson and Marrelec, 2008, Greicius et al., 2009, Raichle et al., 2001) and the “Default Self Network” (Qin and Northoff, 2011). The functional connectivity of the PCC and the precuneus has been associated with the severity of impairment in DOC and is also considered a neuroimaging marker to differentiate vegetative state, minimally conscious state, and coma patients from healthy controls (Vanhaudenhuyse et al., 2010). Moreover, our finding is in line with the results of a previous study on severely brain-injured patients “with impaired consciousness” scanned in the very early phase of the disease (<24 h) (Tsai et al., 2014). The authors reported a significantly lower ALFF in patients within a region of the PCC that largely overlaps with the one reported here. This marker appears to be specific for acute patients. Indeed, previous studies have shown that between-group differences in ALFF may also extend outside the posterior cingulate area when considering chronic DOC patients (He et al., 2014, Huang et al., 2014). Taken together, these findings suggest that the resting state signal captured by ALFF may weaken over time and that the functional integrity loss of the low-frequency fluctuation might progressively spread from the PCC to other brain regions, following structural and neurofunctional connections (Cauda et al., 2018). Albeit indirectly, this evidence further supports the prognostic value of our findings, according to which an adequate level of low-frequency fluctuation may represent a significant marker of subsequent consciousness recovery in acute DOC patients.

Beyond these neurofunctional correlates, one crucial aspect of the current study is represented by the relationship observed between the differential autonomic responses for words and pseudo-words (i.e., the SCR delta index) and the spontaneous activity of the PCC: the higher the differential electrodermal response, the higher the residual resting-state activity in the PCC region. This result is in line with previous task-related fMRI evidence of posterior cingulate cortex activity in the comprehension of simple and complex verbal stimuli (Maddock et al., 2003, Whitney et al., 2009). In an fMRI study, Maddock and colleagues (2003) asked healthy participants to evaluate the emotional valence of words presented through headphones. Results have shown that the evaluation of the valence of both unpleasant and pleasant words was associated with significant activation of the PCC bilaterally. The PCC has also been identified as part of a more complex neural system subserving narrative comprehension. For instance, Whitney and colleagues (2009) have shown increased activation of the PCC and precuneus in response to narrative shifts in a story comprehension task, suggesting that the PCC activity would subserve the ability to follow the plot, evaluating and integrating ongoing linguistic information, in order to accept or reject its integration with prior knowledge (Maguire et al., 1999, Mar, 2004, Martín-Loeches et al., 2008, Whitney et al., 2009, Xu et al., 2005). Moreover, the activity of this brain region had already been associated with emotional word processing (Cato et al., 2004).

Intriguingly, the association between the PCC and the SCR signals has been repeatedly reported, albeit seldom discussed in the literature. According to *Neurosynth* (www.neurosynth.org), a toolbox that allows semantically oriented *meta*-analyses of imaging data, at the time of consultation, 91 papers report a significant fMRI effect in the PCC for SCR indices (search term = “Skin Conductance”, Uniformity test and Posterior Probability = 0.75). Accordingly, the preserved functionality of this region may well be a prerequisite or a marker of the vigilance and awareness needed for higher-level cognitive functioning, hence representing a crucial area for consciousness (Vogt and Laureys, 2005). Notably, the disrupted activity and functional connectivity of the posterior cingulate cortex has previously been identified as a marker of DOC (Crone et al., 2015, Herbet et al., 2014). Our findings support the crucial connection between cortical autonomic regulation, autonomic responses to verbal stimuli, and the prediction of functional recovery of consciousness.

Of course, alternative hypotheses should always be considered in the interpretation of new data. One might argue that the physiological response recorded in outcome-positive patients could be considered an automatic sign of stimuli-related salience. Indeed, electrophysiological task-specific preparatory responses can be preserved during sleep (in the absence of awareness) in healthy subjects (Kouider et al., 2014). Accordingly, the salience of the stimuli, rather than implicit cognitive processing of their lexical-semantic content, might have merely triggered the physiological correlate of the distinction between words and pseudo-words. However, in this case, we would have expected to find a significant correlation between the SCR delta value and regions associated with what has been repeatedly defined as a “Salience Network” (Seeley et al., 2007), such as the dorsal anterior cingulate and orbital frontoinsular cortices. Our empirical findings do not support this hypothesis. Furthermore, pseudo-words may be as salient for other reasons and determine as much arousal due to their strange/unexpected nature, as shown by Plourde et al. (2006). Therefore, if the mere “salience” cannot explain our findings, it is striking to observe that emotionally relevant word stimuli can induce sizeable autonomic responses in patients with positive prognostic outcomes.

In conclusion, a relatively straightforward (neuro)physiological index, such as the event-related SCR, was efficiently adopted and combined with the residual power of a local neuronal activity (e.g., fALFF) to optimise the prognosis for acute DOC patients. Although preliminary, this evidence could represent a promising start in developing an easy-to-use marker of recovery of consciousness.

## 5 Limitations of the study

The results reported in this study may have been inevitably influenced by the adoption of the eyes-closed condition during the fMRI scans. As detailed in the methodology section, we asked the participants to keep their eyes closed to mitigate, as much as possible, potential confounding effects associated with the clinical characteristics of the sample of patients, such as the inability of acute patients to keep their eyes open for 10 min. Nevertheless, we acknowledge that the eye-closed condition may lead some participants to fall asleep during the scanning session, causing a higher variability of the responses (Agcaoglu et al., 2019, Patriat et al., 2013, Tagliazucchi and Laufs, 2014). To control for this risk, we interviewed healthy participants after each resting-state fMRI session about their state of wakefulness. None of the healthy participants subjectively reported falling asleep. Concerning the patients’ sample, being in an acute phase of the pathology, when the sleep-wake cycle may not be recovered yet, the exact phase of the cycle was hardly discriminable. This is one of the main challenges of studying patients affected by DOC at an earlier stage.

## Declaration of Competing Interest

The authors declare that they have no known competing financial interests or personal relationships that could have appeared to influence the work reported in this paper.
