# Supplementary material for: Autonomic responses to emotional linguistic stimuli and amplitude of low-frequency fluctuations predict outcome after severe brain injury
Source: Neuroimage Clin. 2020 Jul 21;28:102356. doi: 10.1016/j.nicl.2020.102356 (PMC7397392; doi:10.1016/j.nicl.2020.102356)
Supplement: Supplementary data 1 [file mmc1.docx]

Supplementary Material for:

*Autonomic responses to emotional linguistic stimuli and amplitude of low-frequency fluctuations predict outcome after severe brain injury.*

Salvato, Gerardo^1,2,3^; Berlingeri, Manuela^3,4,5^; De Maio, Gabriele^1^; Curto Francesco^6^; Chieregato, Arturo^6^; Magnani, Francesca Giulia^1,2,3^; Sberna, Maurizio^7^; Rosanova, Mario^8,9^; Paulesu, Eraldo^3,10,11^; Bottini, Gabriella^1,2,3^.

^1^ Cognitive Neuropsychology Centre, ASST “Grande Ospedale Metropolitano” Niguarda, Milano, Italy

^2^ Department of Brain and Behavioral Sciences, University of Pavia, Pavia, Italy

^3^ NeuroMi, Milan Center for Neuroscience, Milano, Italy

^4^ Department of Humanistic Studies, University of Urbino Carlo Bo, Urbino, Italy

^5^ Center of Developmental Neuropsychology, Area Vasta 1, ASUR Marche, Pesaro, Italy

^6^ Department of Neuroresuscitation and Intensive Care, ASST “Grande Ospedale Metropolitano” Niguarda, Milano, Italy

^7^ Department of Neuroradiology, ASST “Grande Ospedale Metropolitano” Niguarda, Milano, Italy

^8^ Department of Biomedical and Clinical Sciences "Luigi Sacco", University of Milano, Italy;

^9^ Fondazione Europea di Ricerca Biomedica, FERB Onlus, Milano, Italy.

^10^ Psychology Department and NeuroMI-Milan Center for Neuroscience, University of Milano-Bicocca, Milan, Italy.

^11^ fMRI Unit, I.R.C.C.S. Galeazzi, Milano, Italy

**Supplementary Table 1**

|  |  | **Clinical features** | | | | | **CRS-R** | | | | | | | **Lesioned brain region (hemisphere) from the CT/MRI scan** |
| --- | --- | --- | --- | --- | --- | --- | --- | --- | --- | --- | --- | --- | --- | --- |
|  | **Sex (age)** | **Intracranial**  **pathology** | **GCS**  **at admission** | **Worst pupils reactivity to the light** | **Outcome at 30 days** | **Time from onset** | **Auditory function** | **Visual function** | **Motor function** | **Oromotor/**  **Verbal function** | **Communication** | **Arousal** | **Total score** |  |
| **Patient 1** | Female (68) | ICH | 4 | bilateral reactivity | **OP** (GOS-E 3) | 11 | 0 | 1 | 1 | 1 | 0 | 1 | 4 | Frontal lobe (bilateral) |
| **Patient 2** | Male (67) | ICH | 7 | bilateral reactivity | **OP** (GOS-E 3) | 5 | 0 | 1 | 1 | 0 | 0 | 1 | 3 | Thalamus and internal capsule (right) |
| **Patient 3** | Female (66) | ICH | 5 | unilateral  reactivity | **ON** (GOS-E 2) | 14 | 0 | 1 | 1 | 1 | 0 | 1 | 4 | Thalamus and Internal Capsule (right) |
| **Patient 4** | Male (68) | TBI | 3 | unilateral  reactivity | **OP** (GOS-E 3) | 22 | 0 | 1 | 1 | 0 | 0 | 1 | 3 | Frontal lobe (left), Temporal lobe (left), Cerebellum (bilateral) |
| **Patient 5** | Male (67) | ICH | 6 | bilateral reactivity | **ON** (GOS-E 1) | 15 | 0 | 0 | 2 | 0 | 0 | 1 | 3 | Temporal lobe (bilateral) Insula (bilateral) |
| **Patient 6** | Female (66) | TBI | 5 | bilateral reactivity | **OP** (GOS-E 3) | 38 | 3 | 3 | 0 | 2 | 2 | 1 | 12 | Frontal lobe (right), Temporal lobe (bilateral) |
| **Patient 7** | Female (63) | ICH | 3 | unilateral  reactivity | **OP**  **(**GOS-E 3) | 10 | 1 | 1 | 2 | 0 | 0 | 1 | 5 | Temporal lobe (left), Parietal lobe (left), Occipital lobe (left) |
| **Patient 8** | Female (77) | ICH | 4 | bilateral reactivity | **ON** (GOS-E 2) | 6 | 0 | 0 | 1 | 0 | 0 | 0 | 1 | Internal Capsule (right) |
| **Patient 9** | Male (68) | TBI | 5 | bilateral reactivity | **OP** (GOS-E 3) | 33 | 1 | 1 | 1 | 0 | 0 | 2 | 5 | Frontal lobe (bilateral) Occipital lobe (right) |
| **Patient 10** | Male (59) | TBI | 11 | bilateral reactivity | **OP** (GOS-E 4) | 22 | 2 | 3 | 5 | 0 | 0 | 2 | 12 | Frontal lobe (bilateral) Temporal lobe (right) Parietal lobe (right) |
| **Patient 11** | Female (54) | SAM | 7 | bilateral reactivity | **OP**  (GOS-E 3) | 16 | 1 | 1 | 1 | 0 | 0 | 2 | 5 | Frontal lobe (right) |
| **Patient 12** | Female (69) | SAM | 4 | bilateral reactivity | **OP** (GOS-E 3) | 11 | 1 | 1 | 1 | 0 | 0 | 2 | 5 | Frontal lobe (right) Internal Capsule (right) |
| **Patient 13** | Male (43) | TBI | 6 | bilateral reactivity | **OP**  (GOS-E 3) | 10 | 0 | 0 | 2 | 0 | 0 | 0 | 2 | Frontal lobe (bilateral) Temporal lobe (right) Corpus Callosum |
| **Patient 14** | Female (54) | CNS INF | 6 | bilateral reactivity | **ON**  (GOS-E 2) | 20 | 0 | 0 | 3 | 0 | 0 | 1 | 4 | Frontal lobe (bilateral) Insula (bilateral) Cerebellum (bilateral) |
| **Patient 15** | Female (69) | ICH | 6 | unilateral  reactivity | **ON** (GOS-E 2) | 33 | 0 | 1 | 0 | 0 | 0 | 1 | 2 | Frontal lobe (left) Insula (left) Temporal lobe (left) Occipital lobe (left) |

GOS-E=Glasgow Outcome Scale Extended; ON= Outcome Negative patients; OP= Outcome Positive patients; CRS-R=Coma Recovery Scale-Revised, CT/MRI: computed tomography/magnetic resonance imaging; ICH=Intracranial haemorrhage; TBI=Traumatic Brain Injury; SAM=Subaracnoid haemorrhage; CNS INF= Central Nervous System Infection; upR= upcoming responsive patient; upUR= upcoming unresponsive patient. Note that CRS-R is relative to the day when SCR and rs-fMRI were performed.

**Supplementary Table 2**

| ***Authors*** | ***Year*** | ***Stimuli*** | ***Patients*** | ***Stage*** | ***Electrodermal activity*** | ***Study type*** |
| --- | --- | --- | --- | --- | --- | --- |
| Schuri & von Cramon | 1979 | High intensity acoustic stimulus  (from Munchner Coma Skala items) | n = 32 poisoned patients | N.S. | SRL and number of SRR | Observational |
| Péréon et al. | 1995 | Electric stimuli | n = 12 stroke patients (7 normal consciousness, 5 altered consciousness) | Acute/Chronic | Event-related SSR | Prognostic |
| Hildebrandt et al. | 1998 | None | n = 22 traumatic brain-injured patients in coma state | Acute | SCL and SCR at rest | Observational |
| Keller et al. | 2007 | Recorded relative’s voice vs  White noise | n = 18 with Glasgow Coma Scale score less than 6 | Chronic | Event-related Skin conductance response and at rest | Observational |
| Wieser et al. | 2010 | 200 acoustic stimuli (85% standard stimuli at 500Hz; 15% deviant stimuli at 1000Hz) | n = 8 with Glasgow Coma Scale score between 6 and 12 | Chronic | Event-related skin conductance response and during 7 minutes of rest | Observational |
| Daltrozzo et al. | 2010 | 10 emotional sounds  10 neutral environmental sounds  20 filler sounds  (form International Affective Digital Sounds database) | n = 13 (12 in coma state; 1 low-responsive) | Acute | Event-related skin conductance response  For peak analysis: skin conductance response peaks between 8 and 16s from stimulus onset  For waveform analysis: skin conductance response waveforms between 8 and 16s from stimulus onset | Observational |
| Luaute´ et al. | 2018 | Preferred music vs  Neutral sounds | n = 11 (5 in vegetative state; 6 in minimally conscious state) | Chronic | Event-related SCL | Observational |

Authors: the first author of each study is reported; Year: publication date; Stimuli: the type of stimuli for each study is reported; Patients: number and features; Stage: stage of the disease of the patients in each study (Acute, Chronic, N.S= not specified); Electrodermal activity: type of recorded response for each study (SRL=Skin Resistance Level, SRR=Skin Resistance Responses, SSR=Sympathetic Skin Response, SCL=Skin Conductance Level, SCR=Skin Conductance Resistance, GSR=Galvanic Skin Response); Study type: observational or prognostic study.

**Supplementary Table 3**

| **Condition** | **Stimulus** |
| --- | --- |
| *Words* | “(cancro) cancer” “(angoscia) distressed” “(disprezzo) hatred” “(guerra) war” “(stress) stress” “(odio) hate” “(rabbia) angry” “(chirurgia) surgery” “(pericolo) danger” “(problema) trouble” “(confuso) confused” “(nervoso) nervous” “(pressione) pressure” “(sopraffatto) overwhelmed” “(nudo) nude” “(laureato) graduate” “(felice) happy” “(bacio) kiss” “(successo) success” “(orgasmo) orgasm” “(romantico) romantic” “(denaro) cash” “(divertimento) fun” “(vittoria) win” “(madre) mother” “(risata) laughter” “(gioia) joy” “(miracolo) miracle” “(amato) loved” “(amore) love” |
| *Non-words* | “lebifle” “comu” “ziacispor” “famuf” “sovolonu” “elscoatra” “toniargirug” “tosueman” “ptaleat” “toinset” “pnidisoi” “tsogoliav” “tegnansta” “litretode” “lacoscive” “lstagofi” “pintodi” “tilanage” “tomerca” “chiocer” “tearipra” “fogu” “gnelalo” “nefistra” “giovillag” “tapian” “pcoam” “lsarecao” “enrttae” “celuclo” |

List of stimuli used in the event-related electrodermal activity experiment.

**Supplementary analysis**

The direct comparison of whole-brain zfALFF between healthy controls and DOC patients showed a significant between-group difference in a cluster centred in the right posterior cingulate cortex (PCC: *x*=6, *y*=-45, *z*=24, *Z-score*=5.0,  *p*<.05 *FWE*-corrected cluster level). Interestingly, none of these effects fell within the lesion overlap map (see Figure 3 of the manuscript), to further suggest that a spurious effect of brain lesions did not bias our findings. Although the data preprocessing would have excluded the patients' brain lesions in the comparison HC versus DOC patients when isolating the voxel-wise between-groups differences in the level of zfALFF, we also performed an additional analysis to exclude these lesioned areas explicitly. We subtracted the patients’ lesion map from the mask created by SPM when computing the two-samples t-test analysis. We then used the new mask, i.e., a brain volume in which the lesioned areas were excluded, to compare HC and DOC patients in a new analysis. The direct comparison between healthy controls and DOC patients taking into account the preserved brain regions only confirmed the significant between-group difference in a cluster centred in the right posterior cingulate cortex (PCC: x=6, y=-45, z=24; *p*<0.05 *FWE*-corrected cluster level).
